# Plasma levels of mitochondrial and nuclear DNA in patients with massive pulmonary embolism in the emergency department: a prospective cohort study

**DOI:** 10.1186/cc12735

**Published:** 2013-05-24

**Authors:** Francisco Arnalich, Maria Constanza Maldifassi, Enrique Ciria, Rosa Codoceo, Jaime Renart, Carmen Fernández-Capitán, Rafael Herruzo, Francisco Garcia-Rio, Eduardo López-Collazo, Carmen Montiel

**Affiliations:** 1 Department of Internal Medicine and Emergency Medicine Service, Hospital Universitario La Paz. IdiPAZ. Paseo de la Castellana 261.28046 Madrid, Spain; 2 Department of Pharmacology and Therapeutics, Facultad de Medicina Universidad Autónoma de Madrid. IdiPAZ. Arzobispo Morcillo, 4. 28029 Madrid, Spain; 3 Clinical Biochemistry Service, Hospital Universitario La Paz. IdiPAZ. Paseo de la Castellana 261.28046 Madrid, Spain; 4 Biomedical Research Institute "Alberto Sols," Consejo Superior de Investigaciones Científicas-Universidad Autónoma de Madrid. IdiPAZ. Arturo Duperier, 4. 28029 Madrid, Spain; 5 Preventive Medicine Service; 6 Research Unit, Hospital Universitario La Paz. IdiPAZ. Paseo de la Castellana 261.28046 Madrid, Spain

**Keywords:** plasma mitochondrial DNA, plasma nuclear DNA, cell-free plasma DNA, heart-type fatty acid-binding protein, hospital mortality, massive pulmonary embolism, prognosis

## Abstract

**Introduction:**

Cell-free plasma mitochondrial DNA (mt-DNA) and nuclear DNA (n-DNA) are biomarkers with prognostic utility in conditions associated with a high rate of cell death. This exploratory study aimed to determine the plasma levels of both nucleic acids in patients with massive and submassive pulmonary embolism (PE) and to compare them with other biomarkers, such as heart-type fatty acid-binding protein (H-FABP) and troponin I (Tn-I)

**Methods:**

This was a prospective observational study of 37 consecutive patients with massive PE, 37 patients with submassive PE, and 37 healthy subjects. Quantifications of plasma mt-DNA and n-DNA with real-time quantitative polymerase chain reaction (PCR), and plasma H-FABP and Tn-I by commercial assays, were done on blood samples drawn within 4 hours after presentation at the emergency department.

**Results:**

Plasma mt-DNA and n-DNA concentrations were much higher in patients with massive PE (median, 2,970 GE/ml; interquartile range (IQR), 1,050 to 5,485; and 3,325 GE/ml, IQR: 1,080 to 5,790, respectively) than in patients with submassive PE (870 GE/ml and 1,245 GE/ml, respectively; *P *< 0.01) or controls (185 GE/ml and 520 GE/ml, respectively). Eighteen patients with massive PE died of a PE-related cause by day 15 of observation. Plasma mt-DNA and n-DNA values were 2.3-fold and 1.9-fold higher in the subgroup of nonsurviving patients than in survivors. H-FABP and Tn-I values were also higher in patients with massive PE who died (7.3 ng/ml and 0.023 ng/ml, respectively) than in those who survived (6.4 ng/ml, and 0.016 ng/ml, respectively). By receiver operating curve (ROC) analysis, the best cutoff values for predicting 15-day mortality were 3,380 GE/ml for mt-DNA, 6.8 ng/ml for H-FABP, 3,625 GE/ml for n-DNA, and 0.020 ng/ml for Tn-I, based on the calculated areas under the curve (AUCs) of 0.89 (95% confidence interval (CI), 0.78 to 0.99), 0.76 (95% CI, 0.69 to 093), 0.73 (95% CI, 0.58 to 0.91), and 0.59 (95% CI, 0.41 to 0.79), respectively. By stepwise logistic regression, a plasma mt-DNA concentration greater than 3,380 GE/ml (adjusted odds ratio (OR), 8.22; 95% CI, 1.72 to 39.18; *P *< 0.001) and a plasma value of H-FBAP >6.8 ng/ml (OR, 5.36; 95% CI, 1.06 to 27.08; *P *< 0.01) were the only independent predictors of mortality.

**Conclusions:**

mt-DNA and H-FBAP might be promising markers for predicting 15-day mortality in massive PE, with mt-DNA having better prognostic accuracy.

## Introduction

Massive pulmonary embolism (PE) is an infrequent cardiovascular emergency that carries a very high mortality rate [1,2]. Assessment of right ventricular (RV) function and measurement of cardiac biomarkers (troponins, brain natriuretic peptide (BNP), or heart-type fatty acid-binding protein (H-FABP)) released to serum from the RV, accurately predict 30-day mortality in patients with submassive PE [3-7]. Unfortunately, no data are yet available to support the usefulness of cardiac biomarkers in predicting mortality in patients with massive PE [4].

Cell-free plasma DNA comprises small DNA fragments derived from nuclear or mitochondrial double-stranded molecules that are found at very low concentrations circulating in peripheral blood from healthy people. Both nuclear DNA (n-DNA) and mitochondrial DNA (mt-DNA) are released into the circulation from apoptotic and necrotic cells, although the exact mechanism is unclear [8,9]. Cell-free plasma n-DNA and mt-DNA are biomarkers with prognostic utility in a range of conditions associated with a high rate of cell death, including trauma [10], stroke [11], myocardial infarction [12], burns [13], sepsis, and critical conditions [14-16]. We and others reported that high concentrations of plasma n-DNA can predict treatment outcomes and mortality in conditions associated with global ischemia-reperfusion injury, such as acute mesenteric ischemia and cardiac arrest [17-19]. High plasma concentrations of both mt-DNA and n-DNA have been found in patients with acute ischemic stroke, bacterial meningitis, and sepsis [20-22], and with similar prognostic values. In contrast, recent data indicate that increased mt-DNA concentration is a more powerful prognostic marker than plasma n-DNA in patients with out-of-hospital cardiac arrest [23].

Detection of plasma DNA with counterimmunoelectrophoresis was proposed as a screening test for the diagnosis of PE [24]. One study conducted two decades ago showed that increased plasma DNA concentrations had a sensitivity of 82% and a specificity of 85% in detecting patients with PE [25], but no subsequent study evaluated the potential prognostic value in patients with high-risk PE. Examining this question is relevant because those patients have widespread tissue hypoxia that decreases oxygen availability for mitochondria, leading to a rapid depletion of ATP synthesis and cell death via the intrinsic pathway of apoptosis [26]. We hypothesized that the amount of plasma mt-DNA, released to the circulation from apoptotic cells, could be a good new marker of cell damage caused by acute RV myocardial ischemia in patients with massive PE.

This exploratory study aimed to determine the plasma levels of both nucleic acids in patients with massive and submassive PE presenting at the emergency department (ED) and to compare them with other biomarkers, such as heart-type fatty acid-binding protein (H-FABP) and troponin I (Tn-I).

## Materials and methods

### Patients and setting

We performed a prospective observational study of 74 consecutive patients admitted to the ED with acute massive or submassive PE, confirmed with computed tomographic pulmonary angiography (CTPA), who were classified into two groups: 37 patients with massive PE and 37 patients with submassive PE, based on their systolic blood pressure on admission and the presence of echocardiographic findings of right ventricular (RV) dysfunction. The study was conducted from January 2005 through May 2007 at La Paz University Hospital in Madrid, Spain, an academic tertiary care hospital with annual ED visits of around 120,000. Massive PE was diagnosed in patients who met the following criteria: (a) systolic blood pressure <90 mm Hg or a pressure decrease of ≥40 mm Hg for >15 minutes at arriving in the ED, requiring catecholamine administration to maintain adequate organ perfusion, if not due to new-onset arrhythmia, hypovolemia, sepsis, acute coronary syndrome, or acute left ventricular failure; (b) partial oxygen arterial pressure ≤80 mm Hg while receiving supplemental oxygen of 2 L/min, in the absence of pulmonary infiltrates on chest radiograph film.

Submassive PE was diagnosed in patients who were normotensive on admission and had echocardiographic signs of RV dysfunction (see Additional file 1, methods). Exclusion criteria were (a) end-stage heart or renal failure, (b) clinical conditions that might be associated with increased plasma DNA concentrations (metastatic cancer, multiple trauma, stroke, severe sepsis or septic shock, acute myocardial infarction, cardiac arrest). Sample size of the massive-PE group was estimated based on our previous studies in patients with out-of-hospital cardiac arrest, showing that plasma DNA concentrations were at least 1.5-fold higher in patients who died than in survivors [18,23]. Assuming a mortality rate between 30% and 50% in patients with massive PE, and that the magnitude of differences in plasma nucleic acids between survivors and nonsurvivors would be similar in massive PE, we estimated that a sample size of 37 patients would be sufficient to keep a type I (α) error of 0.05 and a type II (β) error of 0.20. The submassive-PE group consisted of the same number of patients, matched in age and gender to the massive-PE group. Each patient´s baseline characteristic determined their risk classification, according to the predictive pulmonary severity index (PESI) model [27]. Thirty-seven healthy community subjects or relatives of patients taking no medications, with a normal physical and analytic check-up, were recruited as the control group. The study protocol was approved by the local research ethics committee. All study and controls subjects or their legal designees signed a written informed consent.

The end point of the study was 15-day PE-related death. Cause of death was judged to be definitive fatal PE, if it was confirmed by autopsy. Possible fatal PE was considered in a patient who died suddenly or unexpectedly of severe PE in the absence of any alternative cause. All patients received standard anticoagulation therapy with intravenous unfractionated heparin or subcutaneous low-molecular-weight heparin. Thrombolytic therapy was strongly considered in patients with massive PE. However, mainly because of high-risk bleeding (six cases) or of the patient legal designee´s refusal to allow thrombolysis (nine cases), only 20 patients were actually given thrombolysis (2-hour infusion of 100 mg recombinant tissue plasminogen activator).

### Blood sampling, processing of plasma, and DNA extraction

A 10-ml blood sample was taken within approximately 4 hours (range, 1 to 7 hours) from admission to the ED, either before heparin administration or less than 2 hours after heparin administration, during the diagnostic workup. After confirmation of the diagnosis, patients or their relatives were asked to authorize the use of thrombolytic therapy and to sign the informed consent form for enrollment into the study. Therefore, in all the cases, blood was obtained before thrombolysis. We prepared and measured plasma DNA by the same methods we used previously [17,18,23]. Plasma and cells were separated by centrifugation at 1,800 *g *(+4ºC) for 10 minutes, and plasma samples were stored at -80ºC. Afterward, plasma was carefully removed from tubes without shaking the buffy coat, transferred to plain polypropylene tubes, and centrifuged at 16,000 *g *(+4ºC) for 10 minutes. The supernatants were collected into fresh polypropylene tubes and stored at -80ºC until further processing. Cell-free DNA was extracted from 200-μl plasma samples by using the QIAamp DNA Blood Mini Kit (Qiagen, Hidden, Germany), according to the manufacturer's recommendations.

### Real-time quantitative PCR

Cell-free plasma DNA copy numbers were measured with real-time quantitative PCR assay (Roche Lightcycler; Roche, Lewes, UK) by using specific primers to amplify the *β-globin *and *MT-ND2 *genes [28,29]. Although the first gene is present in all nucleated cells of the body, the second is unique to mitochondria. An SYBR green-based assay for amplicon detection on the ABI Prism 7900 device was used, as previously described [28]. The following primers were designed for the *β-globin *gene (GenBank accession number U01317): 354**-**forward (5′-GTGCACCTGACTCCTGAGGAGA-3′) and 455**-**reverse (5′-CCTTGATACCAACCTGCCCAG-3′), and for the *MT-ND2 *gene (GenBank accession number NC012920): 156**-**forward (5′-CACAGA AGCTGCCAT CAAGTA-3′) and 245-reverse (5′-CCGGAGAGTATATTGTTGAAGAG-3′). Two amplicons of 101-bp (n-DNA) and 90-bp (mt-DNA) were obtained, respectively, in each of these PCR reactions. A 10-fold serial dilution of human genomic DNA (Roche) was used to construct the calibration curve, which was included in each PCR reaction. The imprecision of this experimental approach was previously reported [28,29], with an interassay CV of the threshold cycle (Ct) values ranging from 0.5% to 1.1%. Results are expressed as genome equivalents (GE)/ml plasma (1 GE = 6.6 pg DNA), with a detection limit of 12.5 GE/ml.

### Laboratory measurements

The plasma levels of different biomarkers were measured as follows: (a) D-dimer by immunoturbidimetric assay (INNOVANCE D-Dimer assay; Siemens Healthcare Diagnostics Products GmbH, Marburg, Germany); (b) Tn-I and NT-proBNP with quantitative electrochemiluminescence immunoassays (Dimension Vista System analyzer; Siemens Healthcare Diagnostics, DE, USA); (c) H-FABP (dilution 1:5) by a solid-phase enzyme-linked immunoadsorbent assay (HyCult Biotechnology, Uden, The Netherlands); (d) markers involved in Fas-related apoptosis, human soluble Fas (sFas), and soluble Fas ligand (sFasL) molecules, with enzyme immunoassay (Quantikine; R&D Systems, Minneapolis, MN, USA) [see Additional file 1, methods]. All other laboratory analyses were performed with standard techniques. Renal insufficiency was defined as a glomerular filtration rate <60 ml/min/1.73 m^2^. The glomerular filtration rate was calculated by using the abbreviated Modification of Diet in Renal Disease formula [31].

### Statistical analysis

Continuous variables are expressed as medians and interquartile ranges (IQR, 25^th ^to 75^th ^percentiles) and compared with the Mann-Whitney *U *test. Categoric variables were given as absolute values and percentages and compared with χ^2^, by using the Fisher exact test when appropriate. We determined bivariate relations by using nonparametric Spearman rank correlation. Receiver operating characteristic (ROC) curves were constructed to determine the optimal cutoff values for maximal sensitivity and specificity of different laboratory markers for predicting the primary end point of 30-day mortality. Comparison of AUCs was performed as recommended by DeLong *et al. *[32]. The results are presented as odds ratio (ORs) with the corresponding 95% CIs. Kaplan-Meier analysis was performed for cumulative survival, and log-rank values were used to assess statistical significance. All significant variables were then tested in a multivariable logistic regression analysis model by using forward variable selection to identify factors that had independent predictive value for 15-day mortality. Statistical significance was set at *P *< 0.05 in all tests. All statistical analyses were performed by using the SPSS for Windows 15.0 (SPSS Inc, Chicago, IL, USA) and MedCalc 9.6.4.0 (MedCalc Sofware).

## Results

Eighteen (48.6%) patients with massive PE died by day 15 of observation, five of them during the first 48 hours. Ten (58.8%) deaths occurred in the group of 17 patients who did not receive thrombolysis, and eight (40.0%) deaths among the 20 patients after thrombolysis. Three patients died of PE confirmed at autopsy. Thirteen PE-related deaths were caused by irreversible RV dysfunction in the absence of any alternative diagnosis. In four other patients, a major hemorrhage probably contributed to death. The median duration of the ICU stay was 14 days (IQR, 5 to 20), and the median time until hospital discharge for survivors was 32 days (IQR, 20 to 51). By contrast, in the submassive-PE group, no deaths occurred, and only five patients required ICU admission (median stay, 4 days). As shown in Table 1, demographics and predisposing risk factors were not significantly different between patients with massive and submassive PE or between patients with massive PE who died and those who survived. Patients´ symptoms and physical findings at presentation were similar in survivors and nonsurvivors. The proportion of patients with PESI (pulmonary embolism severity index) risk classes V and the presence of obstructive shock were significantly higher in nonsurvivors than in survivors. Table 2 displays baseline plasma concentrations of cardiac markers, soluble apoptosis-signaling molecules, mt-DNA, and n-DNA in the three study subjects groups. Statistically significant differences were observed among these variables when all groups were compared (Kruskal-Wallis test; *P *< 0.01). Significantly higher values of all parameters were observed in submassive and massive PE compared with controls, as well as in the submassive-PE group compared with massive-PE group (Mann-Whitney *U *test, corrected by Bonferroni; *P *< 0.01). With regard to the massive-PE group, the median cell-free plasma mt-DNA and n-DNA concentrations at admission were higher in nonsurvivors (4,220 GE/ml and 4,450 GE/ml, respectively) than in survivors (1,830 GE/ml and 2,285 GE/ml, respectively; *P *< 0.01). Significantly higher plasma H-FABP and Tn-I levels, but not significant differences in NT-proBNP concentrations, were noted in patients who died compared with survivors. A significant increase in sFas levels was found in nonsurvivors. The baseline characteristics of massive-PE patients who did and did not receive thrombolysis were similar, except for the fact that thrombolysis patients had significantly higher acidosis and hyperlactatemia. No significant differences were found in plasma concentrations of cardiac markers, apoptosis-signaling molecules, or nucleic acids between both patient subgroups (see Additional file 2, Table S1). Total point scores of PESI were not significantly associated with plasma concentrations of any of these markers. Significant correlations were found between plasma mt-DNA and n-DNA concentrations (*r *= 0.568; *P *< 0.01), and between each of them and H-FABP values (*r *= 0.476 and *r *= 0.412, respectively; *P *< 0.01). Plasma mt-DNA levels also correlated with lactate concentrations (*r *= 0.451; *P *< 0.01) and with sFas value (*r *= 0.379; *P *< 0.01). In contrast, no significant correlation was noted between plasma n-DNA and lactate (*r *= 0.180; *P *= 0.115) or sFas values (*r *= 0.077; *P *= 0.624). Neither mt-DNA nor n-DNA concentrations correlated significantly with creatinine clearance.

**Table 1:** Descriptive characteristic of the study groups

		***Submassive***	**Massive Pulmonary Embolism**			**P value**	**P value**	**Healthy controls**
		***P.Embolism*** ***(n= 37)***	**Total** **(*n *= 37)**	**Survivors** **(*n *= 19)**	**Non-survivors** **(*n *= 18)**	**A**	**B**	**(*n *= 37)**
**Demographics**	Age	*67 (58-73)*	68 (59-74)	66 (59-72)	70 (60-74)	NS	NS	65 (58-74)
	Female	*26 (70.2)*	26 (70.2)	12 (63.1)	*14 (77.7)*	*NS*	*NS*	24 868.6)
**Risk factors**	Obesity (BMI >30)	*12 (32.4)*	14 (37.8)	6 (31.5)	*8 (44.4)*	*NS*	*NS*	
	Immobilization (bed rest)	*6 (16.2)*	8 (21.6)	3 (15.8)	*5 (27.7)*	*Ns*	*NS*	
	Recent major surgery (< 30 d)	*6 (16.2)*	9 (24.3)	4 (21.1)	*5 (27.7)*	*NS*	*NS*	
	Chronic heart failure	*10 (27.0)*	12 (32.4)	5 (26.3)	*7 (38.9)*	*NS*	*NS*	
	COPD/emphysema	*7 (18.9)*	8 (21.6)	4 (21.1)	*4 (22.2)*	*NS*	*NS*	
	Cancer (inactive)	3 (8.1)	5 (13.5)	2 (10.5)	3 (16.6)	NS	NS	
	Previous DVT	6 (16.2)	7 (18.9)	4 (21.1)	3 (16.6)	NS	NS	
	Concurrent DVT	10 (27.0)	14 (37.8)	6 (31.5)	8 (44.4)	NS	NS	
	Unknown	14 (37.8)	13 (35.1)	7 (36.8)	6 (33.3)	NS	NS	
**Clinical data**	Acute onset of dyspnea	21 (56.7)	31 (83.8)	16 (84.2)	15 (83.3)	<0.01	NS	
	Acute chest pain	9 (24.3)	13 (35.1)	6 (31.5)	7 (38.9)	NS	NS	
	Preceding syncope	3 (8.1)	15 (40.5)	7 (36.8)	8 (44.4)	<0.01	NS	
	Systolic blood pressure, mm Hg	119 (113-128)	74 (68-83)	75 (69-82)	73 (67-83)	<0.01	NS	125 (118-134)
	Mean blood pressure, mm Hg	83 (77-91)	46 (38-54)	45 (39-54)	44 (38-52)	<0.01	NS	86 (78-95)
	PaO_2_ mm Hg	93 (90-97)	67 (64-72)	68 (64-73)	66 (63-72)	<0.01	NS	95 (91-98)
	PESI risk class III	11 (29.7)	0 (0.0)	0 (0.0)	0 (0.0)	<0.01	NS	
	PESI risk class IV	20 (70.3)	16 (43.2)	9 (56.2)	7 (43.8)	<0.01	NS	
	PESI risk class V	6 (16.2)	21 (56.8)	8 (38.1)	13 (61.9)	<0.01	<0.05	
**Specific treatment**	Thrombolysis (within 24 h)	0	20 (54.5)	11 (57.9)	9 (50.0)	---	NS	
	Caval filter implantation	0	2 (4.4)	1 (5.3)	1 (5.5)	---	NS	
**Complications/outcome**	Obstructive shock (within 24 h)	2 (5.4)	18 (48.6)	6 (31.6)	12 (66.6)	<0.01	<0.01	
	Mechanical ventilation	0	10 (27.0)	4 (21.1)	6 (33.3)	<0.01	NS	
	Major bleeding	0	4 (10.8)	2 (10.5)	2 (11.1)	---	NS	
**Laboratory values**	pH	7.35 (7.32-7.38)	7.23 (7.19-7.27)	7.25 (7.18-7.31)	7.20 (7.16-7.27)	<0.01	<0.05	7.38 (7.35-7.40)
	Basal lactate (mmol/l)	2.2 (1.6-2.7)	5.7 (4.5-7.3)	4.9 (4.5-6.1)	6.4 (5.7-7.3)	<0.01	<0.05	1.8 (1.3-2.1)
	Estimated GFR (ml/min/1.73 m^2^)	69 (65-73)	65 (61-73)	67 (63-73)	63 (61-67)	<0.01	NS	71 (66-75)

Data are median (IQR) or number (%); **A: **comparison of massive vs. submassive group.; **B: **comparison of survivors vs. non-survivors.

**Table 2:** Comparison of factors associated with 15-day mortality.

	**Healthy controls** (*n *= 37)	**Submassive PE** (*n *= 37)	**Massive** **PE** (*n *= 37)	**Mann-Whitney U-test** **(Massive *vs *Submassive PE)**	**Massive PE** **Survivors** (*n *= 19)	**Massive PE** **Non-survivors** (*n *= 18)	**Mann-Whitney U-test** **(Non-survivors *vs *Survivors)**	
**Troponin I (ng/ml)**	0.003 (0.001-0.005)	0.009 (0.006-0.012)	0.019 (0.012-0.028)	-3.040 *p *<0.002	0.016 (0.012-0.023)	0.023 (0.014-0.28)	-2.042 *p *<0.04	
**D- dimer (μg/ml)**	0.5 (0.1-0.8)	7.8 (4.1-10.4)	12.6 (8.2-15.8)	-2.309 *p *<0.021	11.9 (8.2-15.3)	13.7 (10.6-15.8)	-1.004 NS	
**NT-proBNP (pg/ml)**	235 (130-350)	570 355-840	1720 (770-3185)	-2.750 *p *<0.006	1510 (770-2140)	1940 (950-3185)	-1.332 NS	
**H-FBAP (ng/ml)**	1.4 (0.8-2.1)	3.1 (1.6-4.2)	6.8 (5.7-7.8)	-3.520 *p *<0.001	6.4 (5.7-7.4)	7.3 (6.1-7.8)	-2.839 *p *<0.05	
**Plasma n- DNA (GE/ml)**	520 (230-760)	1245 (680-2315)	3325 (1080-5790)	-3.039 *p *<0.002	2285 (1280-4010)	4450 (1130-5780)	-2.750 *p *<0.016	
**Plasma mt- DNA (GE/ml)**	185 (70-430)	870 (365-1690)	2970 (1050-5485)	-4.151 *p *<0.000	1830 (1050-3215)	4220 (2110-5510)	-3.769 *p *<0.001	
**Plasma sFas (pg/ml)**	1070 (530-1650)	3355 (640-6720)	8940 (5330-13270)	-2.408 *p *<0.013	7680 (6330-9400)	10570 (9050-12870)	-2.355 *p *<0.019	
**Plasma sFasL (pg/ml)**	460 (355-540)	510 (285-580)	790 (570-920)	-1.265 NS	755 (570-845)	810 (640-920)	-1.265 NS	

Data are median (IQR);

COPD: chronic obstructive pulmonary disease; DVP: deep venous thrombosis; pro-BNP: pro-Brain Natriuretic Peptide; H-FBAP: heart-type fatty acid-binding protein; sFas: soluble Fas molecule; sFasL: soluble Fas ligand molecule.

**Figure 1:**
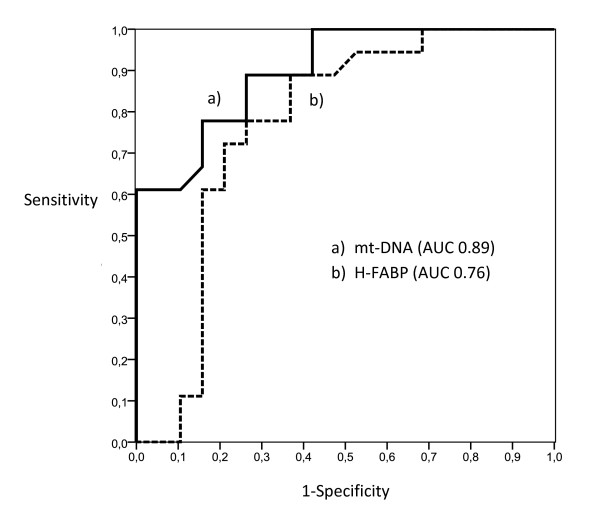
**ROC curves for the use of plasma mt-DNA, and H-FBAP concentrations to predict 15-day mortality**. Plasma mt-DNA had better predictive value (AUC, 0.89; 95% CI, 0.78 to 0.99) than did plasma H-FBAP (AUC, 0.76; 95% CI, 0.69 to 0.93). The best cut-off value of plasma mt-DNA at admission was 3,380 GE/ml (sensitivity, 94.4%; specificity, 68.4%). The best cut-off value of plasma H-FBAP was 6.8 ng/ml (sensitivity, 88.9%; specificity, 85.7%).

**Figure 2:**
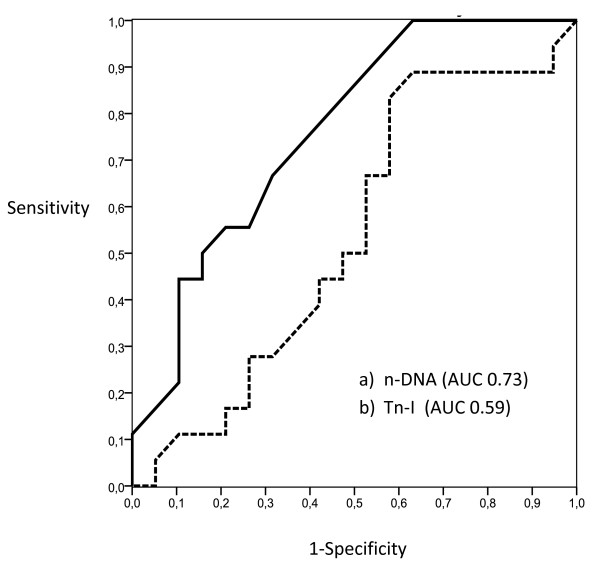
**ROC curves for the use of plasma n-DNA and Tn-I, to predict 15-day mortality**. The best cut-offs were 3,625 GE/ml for n-DNA, and 0.020 ng/ml for Tn-I, based on the calculated AUCs of 0.73 (95% CI, 0.60 to 0.91) and 0.599 (95% CI, 0.41 to 0.79), respectively.

**Figure 3:**
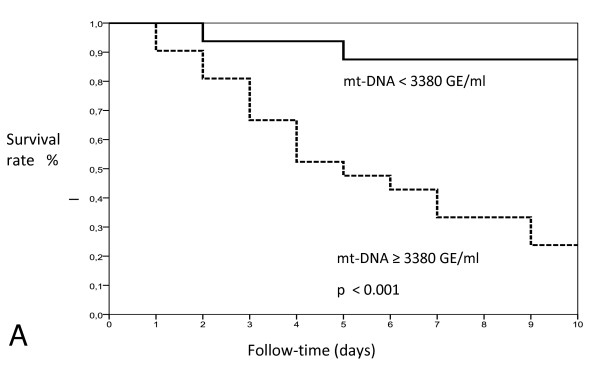
**Kaplan-Meier survival curve analysis according to the plasma concentration of (A) mt-DNA level higher or lower than 3,380 GE/ml; log rank: χ^2^, 13.71; *P *< 0.001**. **(B) H-FBAP **level higher or lower than 6.8 ng/ml; log rank: χ^2^, 12.65; *P *< 0.001. **(C) n-DNA **level higher or lower than 3,625 GE/ml; log rank: χ^2^, 6.84; *P *< 0.009.

ROC curves were calculated for the use of plasma of mt-DNA and n-DNA and two specific cardiac markers, H-FBAP and Tn-I, to predict 15-day mortality (Figures 1 and 2). The best cut-offs were 3,380 GE/ml for mt-DNA, 6.8 ng/ml for H-FABP, 3,625 GE/ml for n-DNA, and 0.020 ng/ml for Tn-I, based on the calculated AUCs of 0.89 (95% CI, 0.78 to 0.99), 0.76 (95% CI, 0.69 to 0.93), 0.73 (95% CI, 0.58 to 0.91), and 0.59 (95% CI, 0.41 to 0.79), respectively (Table 3). The AUC for mt-DNA was similar to that for H-FBAP (*P *= 0.082) but significantly higher than the AUC for n-DNA (*P *= 0.026), and higher than the AUC for Tn-I (*P *= 0.015), in a comparison of all four AUCs, according to DeLong´s method [32]. The discriminant power of these cut-off values (sensitivity, specificity, positive and negative predictive value (PNV, NPV), and likelihood ratio for positive (+LR) and negative (-LR)) are shown in Table 3. Kaplan-Meier survival curves using the best cutoff values of mt-DNA, H-FBAP, and n-DNA are presented in Figure 3 (A through C).

**Table 3:** ROC curves comparing the discriminant power of mt-DNA, H-FBAP, n-DNA and Tn-I to predict 15-day mortality in massive PE.

Test	Cutoff	AUC (95% CI)	**Sens**. (95% CI)	Spec (95% CI)	PPV (%)	NPV (%)	+LR	-LR	*P *value
**mt-DNA**	3380 GE/ml	0.89 (0.78-0.99)	94.4 (82.0-95.6)	68.4 (64.1-87.5)	73.9	92.9	17.6	0.08	<0.001
**H-FBAP**	6.8 ng/ml	0.76 (0.69-0.93)	88.9 (78.3-93.1)	85.7 (76.8-91.8)	69.5	85.7	6.2	0.13	<0.001
**n-DNA**	3625 GE/ml	0.73 (0,58-0.91)	77.8 (64.1-85.5)	57.9 (48.2-71.4)	63.6	73.3	5.03	0.38	<0.05
**Tn-I**	0.020 µg/ml	0.59 (0.41-0.79)	66.7 (51.3-78.0)	47.4 (35.2-60.1)	54.5	60.0	0.75	0.70	NS

We performed multiple logistic regression analysis to identify factors having independent predictive value for mortality. All parameters significantly associated with hospital mortality by univariable analysis (the presence of shock as the only categoric variable, and the serum or plasma concentrations of analytic variables) were included in a multivariable analysis. A plasma mt-DNA concentration >3,380 GE/ml (adjusted OR, 8.22; 95% CI, 1.72 to 39.18; *P *< 0.001) and a plasma value of H-FBAP >6.8 ng/ml (OR, 5.36; 95% CI, 1.06 to 27.08; *P *< 0.01) were the only independent predictors of mortality.

## Discussion

Earlier studies showed detectable plasma DNA in patients with PE, but DNA was measured by using a counter-immunoelectrophoresis technique that yielded unsatisfactory results [24,25]. This is the first prospective observational study to evaluate cell-free plasma mt-DNA and n-DNA concentrations by real-time specific PCR in patients with massive and submassive PE presenting at the ED and to compare them with two specific cardiac markers, H-FABP and Tn-I. We found that plasma concentrations of mt-DNA and n-DNA, H-FABP, and Tn-I were significantly higher in patients with massive PE than in patients with submassive PE. Significantly higher values of these biochemical markers were observed in nonsurviving patients with massive PE than in those who survived. For the prediction of hospital mortality, plasma mt-DNA showed a better discriminative power than that of the other biochemical markers. Furthermore, by multivariable logistic regression analysis, plasma mt-DNA and H-FABP values >3,380 GE/ml and 6.8 ng/ml, respectively, were independent predictors of mortality. However, plasma n-DNA was not an independent predictor and showed less prognostic accuracy.

This report adds new information to existing literature for the study of cell-free plasma circulating nucleic acids in critically ill patients [10-17]. Recently, we and others found elevated plasma levels of cell-free n-DNA in cardiac-arrest patients during the early postresuscitation phase [18,19], a period when reperfusion after whole-body ischemia may trigger a systemic inflammatory response similar to that seen in severe sepsis, causing the release of DNA from damaged cells. Similarly, after massive PE, patients have widespread ischemic tissue damage derived from impaired lung function and abrupt systemic arterial hypotension. When appropriate treatment is started in these patients, the sudden return of oxygen at the onset of reperfusion with restoration of mitochondrial respiration will increase mitochondrial ROS formation to a level that exceeds the antioxidant capacity of the cells [33], causing mitochondria membrane rupture. Thus, it is likely that plasma mt-DNA will be a good new marker of the intensity of cell damage associated with ischemia and reperfusion injury. mt-DNA has been found to be a good early prognostic marker in out-of-hospital cardiac-arrest patients [23]. The current study demonstrates that plasma mt-DNA and n-DNA levels increase shortly after submassive or massive PE, and this elevation can be related to clinical severity and hospital mortality. Elevated mt-DNA is a strong predictor of 15-day mortality in massive PE, and it has better prognostic accuracy than n-DNA. Therefore, in contrast to previous reports testing simultaneously mt-DNA and n-DNA in different conditions [20-22], our data indicate that measurement of both markers is not necessary to increase the prognostic value.

Elevated plasma Tn-I and H-FBAP levels are associated with RV dysfunction and have demonstrated significant prognostic value for risk stratifying in patients with normotensive PE [34,35]. However, their usefulness in the setting of massive PE remains unknown. In the present study, we found a significantly higher plasma concentration of H-FABP in nonsurviving than in surviving patients, but, even so, this marker was not so good a predictor of mortality as was plasma mt-DNA. A plausible explanation is that mt-DNA as a nonspecific global marker of cell death might better reflect the degree of widespread ischemic tissue damage that occurred in massive PE.

Our study showed that the basal lactate concentrations were significantly higher in hospital nonsurvivors than in survivors. Hyperlactatemia in critically ill patients may be attributed to anaerobic glycolysis resulting from inadequate tissue oxygenation, or it may be mediated by inflammation [36]. Excessive lactate production in our patients with acute PE is probably driven by two mechanisms, ischemia-reperfusion injury and the systemic inflammatory response, which both cause severe cell damage. The significant correlation between lactate concentrations and both mt-DNA and n-DNA observed in this study could be a direct consequence of the hypoxia-induced apoptosis and systemic inflammation.

This study provides some evidence regarding the origin of plasma nucleic acids from apoptotic cell death. Apoptosis is generated mainly by the Fas system. Fas (CD95) is a transmembrane protein that transduces a cell-death signal to the cytoplasm on its cross-linking with the Fas ligand (FasL). Hypoxia-induced cell death is known to occur mainly via the mitochondrial (intrinsic) pathway of apoptosis [37], which could explain the stronger relation we found between sFas and mt-DNA. Our results are in line with the findings of histologic analysis of RV muscle from patients with fatal PE that shows accumulation of monocytes/macrophages in the RV, but without histologic evidence of cell necrosis [38]. Also, our findings are in keeping with recent data showing a high plasma cell-free nuclear DNA concentration and apoptotic DNA fragmentation in patients with bacteremia [39]. Further investigation is needed on the association of circulating markers of apoptosis, plasma mt-DNA concentration, and outcome in critical illness, particularly in massive PE.

Some methodologic limitations have to be considered in this study. First, the levels of the plasma nucleic acids may be influenced by age and underlying diseases. However, because no differences in age, predisposing factors, or comorbidities were found in survivors and nonsurvivors, it is likely that differences in plasma mt-DNA and n-DNA levels reflect the acute event of massive PE rather than chronic illness.

Second, excessive accumulation of DNA in plasma may be partly due to a decrease in clearance efficiency. We found that serum urea or creatinine concentrations were not independently associated with plasma mt-DNA and n-DNA concentrations, and this is consistent with data from experimental studies suggesting that renal clearance is not the main mechanism of removal for nucleic acids from the circulation [41-43]. However, further investigations are required to understand the dynamics of plasma DNA removal in patients with impaired renal and hepatic function.

Third, some potential confounders, such as patient management in the emergency department and intensive care unit, are difficult to control.

Fourth, we studied plasma levels only in the emergency department and did not measure levels serially, so we cannot assess the variation of nucleic acid levels in the intensive care unit or over time.

Fifth, our study design allows us to evaluate only associations, and not causes. Opposing these limitations, the strengths of this study lie, first, in the prospective design that includes a clearly defined group of patients with massive and submassive PE. Second, we made a complete recording of predisposing risk factors, clinical and laboratory covariates, and cardiac biomarkers of severity, particularly H-FABP, against which plasma mitochondrial and nuclear DNA may be compared.

Third, it explores the potential association of apoptosis and soluble apoptotic signaling molecules with circulating nucleic acids.

## Conclusions

This study first demonstrates that plasma levels of mt-DNA and n-DNA, and those of H-FABP and Tn-I, were significantly higher in patients with massive PE compared with those with submassive PE. Significantly higher values of all of them were observed in nonsurviving patients with massive PE than in those who survived. For the prediction of hospital mortality, plasma mt-DNA and H-FBAP showed better discriminative power than did n-DNA. The significant association we found between plasma concentrations of both nucleic acids and the indirect marker of apoptosis, sFas, suggests that hypoxia activation of apoptosis may be the main source of plasmatic nucleic acids in PE. Further studies should analyze the relevance of plasma mt-DNA and H-FBAP in rapidly identifying patients with massive PE embolism at the highest risk of mortality who may benefit from catheter-directed therapies if they are unable to receive thrombolytics.

## Key messages

• Plasma mt-DNA and n-DNA concentrations are sensitive global indicator of 15-day mortality in patients arriving at the emergency department with massive PE.

• Plasma mitochondrial DNA concentration in the emergency department was 2 times higher in hospital nonsurvivors than in surviving patients.

• Plasma mt-DNA concentration is more powerful predictor than H-FBAP or n-DNA on admission.

• Both plasma mt-DNA and H-FABP concentrations are strong independent early predictors for hospital mortality, whereas the plasma nuclear DNA seems to have a lower relevance.

• The observed significant association of circulating apoptosis markers sFas with plasma mitochondrial DNA could reflect the anoxia activation of the mitochondrial pathway of apoptosis.

## Abbreviations

AUC: area under the curve; BNP: brain natriuretic peptide; H-FBAP: heart-type fatty acid-binding protein; mt-DNA: mitochondrial DNA; n-DNA: nuclear DNA; ROC: receiver operating curve; RV: right ventricle. sFas: soluble form of Fas molecule; sFasL: soluble form of Fas ligand molecule; Tn-I: troponin I.

## Competing interests

The authors declare that they have no competing interests.

## Authors' contributions

FA, MCM, EC, RC, and CFC contributed to acquisition of the data; FA, JR, FGR, ELC, and CM participated in its design, coordination, and statistical analysis; RC and CM performed the molecular analysis; FA, FGR, ELC, and CM drafted the manuscript. All authors read and approved the final manuscript.

## Supplementary Material

Additional file 1 **METHODS: A. Echocardiography: Transthoracic echocardiography confirmed the presence of RV dysfunction in each case by any of the following parameters: a) RV/LV >0.6 with RV free wall hypokinesis; b) systolic flattening of the interventricular septum; c) elevated tricuspid valve pressure gradient exceeding 30 mm Hg with a shortened acceleration time of pulmonary ejection below 80 m/s in the absence of RV hypertrophy**. **B**. **Normal values and analytical detection limits of the assays: **The normal cutoff value for TnI is 0.08 ng/mL, and 125 pg/ml for NT-proBNP in patients <75 years old or < 450 pg/ml in patients ≥75 years old. The normal cutoff value for H-FABP is <1.6 ng/ml, with a detection limit of 102 pg/ml. The detection limit of markers involved in Fas-related apoptosis, human soluble Fas (sFas) and soluble Fas ligand (sFasL) were 20 pg/ml, with a detection limit of 2 pg/ml. Click here for file

Additional file 2 **Table S1**. Comparison of factors between patients who receive and did not received thrombolysis Click here for file
