# Supplementary material for: Plasma levels of mitochondrial and nuclear DNA in patients with massive pulmonary embolism in the emergency department: a prospective cohort study
Source: Crit Care. 2013 May 24;17(3):R90. doi: 10.1186/cc12735 (PMC3707013; doi:10.1186/cc12735)
Supplement: Additional file 1 — METHODS: A. Echocardiography: Transthoracic echocardiography confirmed the presence of RV dysfunction in each case by any of the following parameters: a) RV/LV >0.6 with RV free wall hypokinesis; b) systolic flattening of the interventricular septum; c) elevated tricuspid valve pressure gradient exceeding 30 mm Hg with a shortened acceleration time of pulmonary ejection below 80 m/s in the absence of RV hypertrophy. B. Normal values and analytical detection limits of the assays: The normal cutoff value for TnI is 0.08 ng/mL, and 125 pg/ml for NT-proBNP in patients <75 years old or < 450 pg/ml in patients ≥75 years old. The normal cutoff value for H-FABP is <1.6 ng/ml, with a detection limit of 102 pg/ml. The detection limit of markers involved in Fas-related apoptosis, human soluble Fas (sFas) and soluble Fas ligand (sFasL) were 20 pg/ml, with a detection limit of 2 pg/ml. [file cc12735-S1.DOC]

**Additional file number 1**

**METHODS:**

1. **Echocardiography :**

Transthoracic echocardiography confirmed the presence of RV dysfunction in each case by any of the following parameters: a) RV/LV >0.6 with RV free wall hypokinesis; b) systolic flattening of the interventricular septum; c) elevated tricuspid valve pressure gradient exceeding 30 mm Hg with a shortened acceleration time of pulmonary ejection below 80 m/s in the absence of RV hypertrophy.

1. **Normal values and analytical detection limits of the assays:**

The normal cutoff value for TnI is 0.08 ng/mL, and 125 pg/ml for NT-proBNP in patients <75 years old or < 450 pg/ml in patients ≥75 years old. The normal cutoff value for H-FABP is <1.6 ng/ml, with a detection limit of 102 pg/ml. The detection limit of markers involved in Fas-related apoptosis, human soluble Fas (sFas) and soluble Fas ligand (sFasL) were 20 pg/ml, with a detection limit of 2 pg/ml.
