# Supplementary material for: Plasma levels of mitochondrial and nuclear DNA in patients with massive pulmonary embolism in the emergency department: a prospective cohort study
Source: Crit Care. 2013 May 24;17(3):R90. doi: 10.1186/cc12735 (PMC3707013; doi:10.1186/cc12735)
Supplement: Additional file 2 — Table S1. Comparison of factors between patients who receive and did not received thrombolysis [file cc12735-S2.DOC]

Table S1. Comparison of factors between patients who receive and did not received

thrombolysis

_______________________________________________________________________

|  | Thrombolysis  (n=20) | No Thrombolysis  (n=17) | p |
| --- | --- | --- | --- |
| **PE related death, 15-day mortality** | 8 (40.0) | 10 (58.8) | NS |
| **Age, years** | 65 (61-70) | 71 (62-74) | NS |
| **Female,** | 13 (65.0) | 13 (76.4) | NS |
| **Obesity (BMI >30)** | 8 (40.0) | 6 (35.3) | NS |
| **Immobilization (bed rest)** | 4 (20.0) | 4 (23.5) | NS |
| **Recent major surgery (< 30 days)** | 3 (15.0) | 6 (35.3) | NS |
| **Chronic heart failure** | 5 (25.0) | 7 (41.2) | NS |
| **COPD/emphysema** | 5 (25.0) | 3 (17.6) | NS |
| **Cancer (inactive)** | 2 (10.0) | 3 (17.6) | NS |
| **Concurrent DVP** | 8 (40.0) | 6 (35.3) | NS |
| **Acute onset of dyspnea** | 17 (85.0) | 14 (82.4) | NS |
| **Acute chest pain** | 8 (40.0) | 5 (29.4) | NS |
| **Preceding syncope** | 9 (45.0) | 6 (35.3) | NS |
| **Systolic blood pressure** | 75 (69-84) | 70 (66-80) | NS |
| **Obstructive shock (within 24 h)** | 7 (35.0) | 11 (64.7) | <0.05 |
| **Mechanical ventilation** | 4 (20.0) | 6 (35.3) | NS |
| **pH** | 7.25 (7.18-7.30) | 7.20 (7.15-7.27) | <0.05 |
| **Basal lactate (mmol/l)** | 6.3 (4.8-7.0) | 5.1 (4.5-7.2) | <0.05 |
| **Estimated GFR (ml/min/1.73 m^2^)** | 63 (53-65) | 56 (49-62) | <0.05 |
| **Troponin I (µg/ml)** | 0.020 (0.012-0.026) | 0.016 (0.012-0.022) | NS |
| **D- dimer (μg/ml)** | 11.8 (8.2-14.0) | 13.1 (10.6-15.8) | NS |
| **NT-proBNP (pg/ml)** | 1565 (670-2860) | 1920 (840-3150) | NS |
| **HFBAP (ng/ml)** | 7.1 (5.5-7.7) | 6.6 (5.3-7.3) | NS |
| **Plasma nuclear DNA (GE/ml)** | 2955 (1280-5340) | 3720 (1130-5845) | NS |
| **Plasma mitocondrial DNA (GE/ml)** | 2640 (1050-5215) | 3485 (2110-5485) | NS |
| **Plasma sFas (pg/ml)** | 8610 (5460-12400) | 9425 (7500-13075) | NS |
| **Plasma sFasL (pg/ml)** | 815 (572-930) | 765 (541-870) | NS |

COPD: chronic obstructive pulmonary disease; DVP: deep venous thrombosis; pro-BNP: pro-Brain Natriuretic Peptide; HFBAP: heart-type fatty acid-binding protein; sFas: soluble Fas molecule; sFasL: soluble Fas ligand molecule.
